# 

**Published:** 1894-02

**Authors:** 


One of the prettiest calendars of the season is issued by the New York Engraving and Printing Co., of 320–322 Pearl street, New York City. It is mounted on a cardboard panel, $7\frac{1}{4}$ x 14 inches, of dark heliotrope color, with margins decorated in silver. The calendar proper is one inch less in dimensions than the panel, and each leaf, besides containing the almanac for the month, displays a photogravure of a prominent actress. The execution of the whole work is exceptionally tasteful, and makes a pretty decoration for a physician's office.

Decidedly the most artistic calendar of the year is published and issued by Messrs. Lee & Shepard, 10 Milk street, Boston. It is entitled, "All Around the Year," and is designed in color by Mrs. Pauline Sunter, whose fame in water colors is world-wide. It is printed on heavy cardboard, with gilt edges, and is equipped with chain, tassels, and ring. Its size is $4\frac{1}{2}$ x $5\frac{1}{2}$ inches, and makes a neat and useful wall decoration when hung by its silvery chain and tied with its dainty white-silk cord. The designs are made up of quaint little figures in grotesque attitudes, that give piquant significance to the verses that they illustrate. Each figure has its own peculiar individuality, that strongly suggests an interpretation of the relationship of figure to word. Furthermore, "All Around the Year" has a calendar for each month of 1894. It makes a tasteful and acceptable gift for a friend, and each comes in a neat box at the nominal price of 50 cents.

The Transactions of the American Association of Obstetricians and Gynecologists, Volume VI., 1893, is now ready for delivery. It is a handsome octavo volume, illustrated, and is printed by William J. Dornan, Philadelphia. Non-members of the Association desiring this book should promptly address the Secretary, Dr. William Warren Potter, 284 Franklin street, Buffalo, as the edition is limited. Price, cloth, $5.00 ; half Russia, $6.00.

The Epitome of Medicine has been discontinued. The publishers, Messrs. G. P. Putnam's Sons, anounce in the December issue that they have decided to consolidate the Epitome with Braithwaite's Retrospect, and they now ask the attention of the readers of the Epitome to the value of the Retrospect as a comprehensive repertory of the medical science of the world.

E. B. TREAT, medical publisher, announces to issue shortly A System of Legal Medicine, a complete work of reference for medical and legal practitioners, by Allan McLane Hamilton, M.D., of New York, and Lawrence Godkin, Esq., of the New York Bar, assisted by thirty collaborators of recognized ability. In two royal octavo volumes of about 700 pages each. Fully illustrated.

The great need of a standard American work on medical jurisprudence has long been felt; and this work gives abundant promise of being just what the medical and legal profession have so long wanted. Every department will be thoroughly and reliably treated.

---

MATHEWS'S MEDICAL QUARTERLY, a journal devoted to diseases of the rectum, gastro-intestinal disease, and rectal and gastro-intestinal surgery, has made its appearance. Its first number, January, 1894, is an exceedingly handsome magazine, standard octavo in size, printed on tinted paper, in long primer type, and contains 188 pages of interesting reading matter. It is owned and edited by Dr. Joseph M. Mathews, professor of surgery and diseases of the rectum in Kentucky School of Medicine, who has associated with him Dr. Henry E. Tuley as assistant editor and manager. It is printed by John P. Morton & Co., Louisville, and will be published on the first of January, April, July and October in each year.

It has no rival in the field that it proposes to occupy, and nothing but success can crown the efforts of its distinguished editor in his useful and masterful enterprise.

We regret that an advance notice of this journal in our January number inadvertently became attached to Mr. E. B. Treat's book announcements.

---

NEW EDITION OF THE NATIONAL DISPENSATORY.—Physicians and pharmacists will be interested to learn the fact that the new edition of *The National Dispensatory* is almost ready for publication. Upon its first appearance fifteen years ago, a very large edition was exhausted in six months. The characteristics which secured this immediate recognition were its authoritative accuracy, its completeness, and the convenience with which desired information could be found owing to the exclusion of obsolete matter. These features have been carefully preserved in the successive editions, of which five have been demanded at brief intervals. The work contains the latest and ripest knowledge of that pharmaceutical savant, the late John M. Maisch, who had practically completed before his death the sections reserved for himself. He had confided the remainder of the pharmaceutical portion to Prof. Charles Caspari, Jr., who occupies the chair of Pharmacy at the Maryland College of Pharmacy, in Baltimore. The therapeutical department has been brought thoroughly abreast of the time by Prof. Alfred Stillé, M. D., who has included critical statements of the value of even the newest remedial agents. A most suggestive "Therapeutical Index" is provided, giving practical suggestions under the various diseases arranged in alphabetical order. This, together with the general index, contains the vast total of 25,000 references. *The National Dispensatory* covers by authorization the new United States Pharmacopeia. Though the new edition of the *Dispensatory* contains at least 100 pages more than its predecessor, it will probably be maintained at the same low price in view of the certainty of a large and growing demand. It contains many new tables, list and descriptions of new processes and tests, and will be a work of indispensable value to all who have to do with any of the medical sciences.

